# Case Report: Vaccine-Induced Immune Thrombotic Thrombocytopenia in a Pancreatic Cancer Patient After Vaccination With Messenger RNA−1273

**DOI:** 10.3389/fmed.2021.772424

**Published:** 2021-11-01

**Authors:** Po-Hsu Su, Yi-Ching Yu, Wen-Hsin Chen, Hsuan-Ching Lin, Yih-Ting Chen, Ming-Huei Cheng, Yen-Min Huang

**Affiliations:** ^1^Division of Hematology and Oncology, Department of Internal Medicine, Hemophilia and Thrombosis Treatment Center, Chang Gung Memorial Hospital at Keelung, Keelung, Taiwan; ^2^Department of Neurology, Chang Gung Memorial Hospital at Keelung, Keelung, Taiwan; ^3^Division of Cardiology, Department of Internal Medicine, Chang Gung Memorial Hospital at Keelung, Keelung, Taiwan; ^4^Division of Nephrology, Department of Internal Medicine, Chang Gung Memorial Hospital at Keelung, Keelung, Taiwan; ^5^Department of Plastic Surgery, Center of Tissue Engineering, Chang Gung Memorial Hospital at Linkou and College of Medicine, Chang Gung University, Taoyuan, Taiwan; ^6^Institute of Medicine, Chung Shan Medical University, Taichung, Taiwan

**Keywords:** messenger RNA-1273, vaccine-induced immune thrombotic thrombocytopenia, pancreatic cancer, COVID-19, platelet factor 4 (PF4)

## Abstract

Vaccination plays an important role during the COVID-19 pandemic. Vaccine-induced thrombotic thrombocytopenia (VITT) is a major adverse effect that could be lethal. For cancer patients, cancer-related thromboembolism is another lethal complication. When cancer patients receive their COVID-19 vaccines, the following thromboembolic events will be more complicated. We presented a case recently diagnosed with pancreatic cancer, who had received the mRNA-1273 (Moderna) vaccination 12 days prior. Ischemic stroke and VITT were also diagnosed. We aggressively treated the patient with steroids, immunoglobulin, and plasma exchange. The titer of anti-platelet factor four and d-dimer level decreased, but the patient ultimately died. The complicated condition of VITT superimposed cancer-related thromboembolism was considered. To our knowledge, only one case of mRNA-1273 related VITT was reported, and this case study was the first to report a cancer patient who was diagnosed with VITT after mRNA-1273 vaccination. Therefore, when the need for vaccination among cancer patients increased under the current COVID-19 pandemic, the possible risk of VITT for cancer patients should be carefully managed. Further studies of the risk evaluation of the COVID-19 vaccine in cancer patients might be required in the future.

## Introduction

The coronavirus disease 2019 (COVID-19) pandemic caused by severe acute respiratory syndrome coronavirus 2 (SARS-CoV-2) had caused many mortalities since 2019. Cancer patients with COVID-19 seem to have a greater mortality risk compared to the general population (1). Thus, vaccination for cancer patients could prevent getting infected with COVID-19 (2).

Several vaccines against SARS-CoV-2 were developed and administrated worldwide, including BNT162b2 (BioNTech/Pfizer), mRNA-1273 (Moderna), Ad26.COV2.S (Johnson & Johnson), and ChAdOx1 (AstraZeneca). An association between the ChAdOx1 and rare cases of vaccine-induced immune thrombotic thrombocytopenia (VITT) or thrombosis with thrombocytopenia syndrome (TTS) was observed (3), with a very low prevalence (4).

Thirteen cases of immune thrombocytopenia were recorded among 16,260,102 doses of the mRNA-1273 vaccine (5). No cerebral venous sinus thrombosis (CVST) was found among 16,471 individuals who received at least one dose of mRNA-1273 by the Mayo Clinic Health System (6). In Germany, no CVST was reported after 1.2 million mRNA-1273 doses (7). Bases on the EudraVigilance database of the European Medical Agency, 213 CVST cases were identified, only one case of mRNA-1273-related CVST showed no thrombocytopenia (8). To our knowledge, only one case of VITT was reported after mRNA-1273 vaccination (9). Here we reported a pancreatic cancer patient who developed VITT after mRNA-1273 vaccination.

## Case Description

A 70-year-old man with atrial fibrillation, chronic obstructive pulmonary disease, and hypertension presented to our hospital with left side weakness for few hours. He had received a first dose of the mRNA-1273 vaccine 7 days before the onset of symptoms. He had regular rivaroxaban 10 mg per day for stroke prevention and had no known prior heparin exposure.

**Figure 1:**
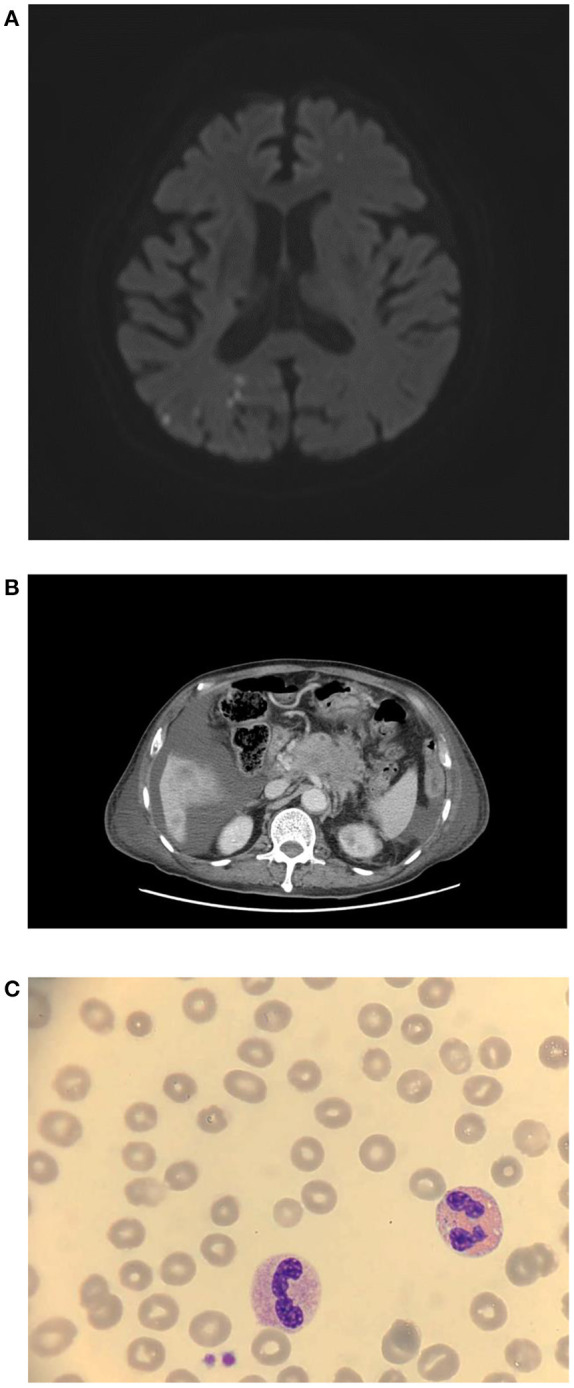
**(A)** Brain MRA revealed diffuse right posterior cerebral territory infarction, 7 days after vaccination. **(B)** Accidentally, stage IV pancreatic cancer was found on abdominal CT. **(C)** Blood smear, 9 days after vaccination.

**Figure 2:**
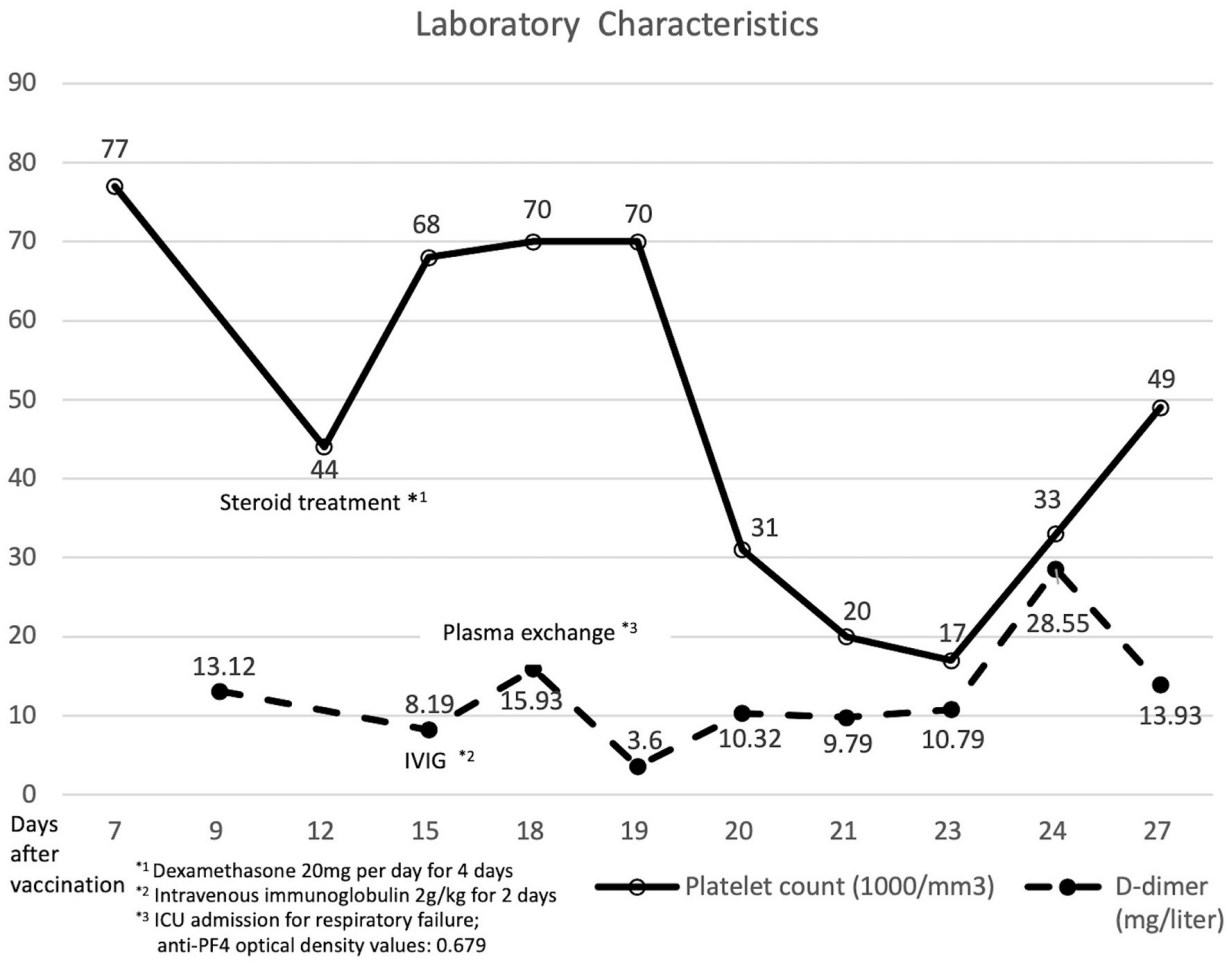
Laboratory data and clinical course of the patient with vaccine-induced immune thrombotic thrombocytopenia.

Creatinine, electrolytes, lactate dehydrogenase, aspartate aminotransferase, and alanine aminotransferase concentrations were within the normal limit. Magnetic resonance angiography of the brain showed scattered infarcts at the right thalamus, parietal cortex, medial temporal, parietal-occipital lobe, and left centrum semiovale (Figure 1A). A posterior cerebral artery territory infarct was also impressed. Moreover, the patient had moderate thrombocytopenia (77 × 10^9^ cells/L) (Table 1, Figure 1C, and Figure 2). Given the thrombocytopenia and medical history, recombinant tissue-type plasminogen activator (rt-PA) or aspirin were not administrated.

**Table 1:** Laboratory characteristics.

**Selected study**	**Result (reference value)**
Polymerase chain reaction test for SARS-CoV19 virus test using a nasopharyngeal swab	virus not detected
Platelet count at admission, *1000/uL*	77,000 (150000–450000)
Platelet count nadir, *1000/uL*	17,000 (150000–450000)
Fibrinogen level nadir, *mg/dl*	63 (200–400)
D-dimer peak, *mg/l*	28.55 (< 0.5)
Prothrombin time at admission, *s*	14.5 (11–12.5)
Mean of standard Prothrombin time at admission, *s*	10.2
The international normalized ratio at admission	1.5(<1.2)
Mixing PT corrected PT, *s*	11.9
Activated partial thromboplastin time at admission, *s*	34.7(25–35)
Mean of standard activated partial thromboplastin time at admission, *s*	28.4
Mixing APTT corrected APTT, *s*	29.1
Thrombin Time, *s*	18.4 (14–21)
Anti-platelet factor 4/polyanion antibody at diagnosis, *optical density unit*, initial	0.679 (<0.399)
Anti-platelet factor 4/polyanion antibody at diagnosis, *optical density unit*, repeat after plasma exchange	0.279 (< 0.399)
C3, *mg/dl*	91.9 (90–180)
C4, *mg/dl*	12.3 (10–40)
Platelet function test, collagen/EPI, *s*	>220 (82–150)
Platelet function test, collagen/ADP, *s*	>255 (62–100)
ANA	negative (≦1:80)
Anti-ds DNA, *WHOunit/mL*	Negative, 12.7(<92.6)
CEA, *ng/mL*	358.0 (Smoker:≦6.5; Nonsmoker: <5.0)
CA199, *U/mL*	16.5 (<27)
Creatinine at admission, *mg/d*L	2.49 (0.64~1.27)
Sodium at admission, *mEq/L*	133 (134~148)
Potassium at admission, *mEq/L*	4.1 (3.6~5.0)
Total bilirubin at admission, *mg/d*L	1.4 (≦1.2)
ALT/GPT, *U/L*	55 (<36)
Albumin at admission, *g/dL*	2.87 (3.5–5.5)

Then, 12 days after vaccination, his thrombocytopenia progressed (44 × 10^9^ cells/L) and his d-dimer level elevated (13.12 mg/L; reference value, <0.55 mg/L). The fibrinogen level decreased mildly (183 mg/dl; reference value, 190–380 mg/dl). The enzyme-linked immunosorbent assay for anti-platelet factor 4 (PF4) polyanion antibody was positive (0.679 optical density; reference value, <0.4 optical density). The diagnosis of VITT was confirmed.

Twenty milligrams of dexamethasone for 4 days and 2 gm/kg/day of immunoglobulin for two consecutive days were administered intravenously. After intravenous immunoglobulin, his platelet counts gradually recovered (68 × 10^9^ cells/L), and the d-dimer level decreased (8.19 mg/L). The patient had progressive dyspnea and a lower fibrinogen level (63 mg/dl). A computed tomography angiogram of the chest showed no evident pulmonary embolism but bilateral pleural effusion and massive ascites. Pancreatic cancer with multiple liver metastases was found (Figure 1B) accidentally, which was compatible with elevated CEA (358.0 ng/mL) and CA-199 (16.5 U/ml) levels. We initiated diuretics for fluid overload. Three days later, plasma exchange was also performed to further increase the d-dimer level (15.93 mg/L). However, the patient continued to deteriorate with respiratory distress; thus, endotracheal intubation with ventilator support was performed.

The d-dimer level and platelet count fluctuated even after plasma exchange and steroid administration. The repeated anti-PF4 antibody level was decreased (0.279 optical density). Panendoscopy was performed for hemostasis of massive upper gastrointestinal tract bleeding, which showed diffuse hemorrhage over gastric mucosa. Uncontrolled atrial fibrillation was recorded. Twenty-eight days after vaccination, the patient had sudden onset of cardiac arrest and was finally expired.

## Discussion

For VITT cases, thrombotic thrombocytopenic purpura (TTP) was a differential diagnosis. Several case reports had mentioned the development of TTP after vaccination, including BNT162b2 (10, 11), ChAdOx1 (12), and Ad26.COV2-S (13). Diagnosis of TTP typically correlates with severely reduced ADAMTS13 activity. In addition, the presence of anti–PF4 IgG supports the diagnosis of VITT. In our case, the absence of schistocytes or microangiopathic hemolytic anemia on the blood smear excluded the possibility of TTP. The patient developed the VITT 12 days after vaccination of mRNA-1273 with cerebral arterial thrombosis, thrombocytopenia, markedly elevation of d-dimer, and positive ELISA on anti-PF4 antibody, thereby fulfilling the American Society of Hematology criteria for VITT. Urgent medical evaluation for VITT is indicated, usually 4–42 days after vaccination (14). Both venous and arterial thromboses have been described, whereas cerebral venous thrombosis (CVST) was considered the most common site in some reports (3, 4).

The pathophysiology of VITT had been discussed in several studies (15, 16). All these reported VITT patients had high levels of IgG antibodies against the complex formed by PF4 and the heparin analog poly (vinyl sulfonate). This finding led to platelet activation and aggregation, which caused thrombosis and platelet consumption. Referring to previous VITT experience with the adenovirus-carrier vaccine, we initiated steroid treatment when the diagnosis was diagnosed. IVIG was administrated immediately after the positive finding of the anti-PF4 polyanion antibody. The patient had pulmonary edema 2 days after immunoglobulin administration. Transfusion-related immune response, fluid overload, or deterioration of heart function could be the possible etiology. The anti-PF4 activities decreased, but progressed thrombus formation was another consideration.

Plasma exchange could be a potential method for clearing autoantibodies (17). Some experts view plasma exchange as a plausible rescue therapy in depleting inflammatory mediators and extracellular vesicles (18). In our case, the d-dimer level dropped, and the platelet count initially improved after plasma exchange. However, the d-dimer level recovered, and the platelet count became worse after other plasma exchanges.

Trousseau's syndrome, the hypercoagulable state associated with malignancy, was a possible explanation for the poor response (19). Pancreatic cancer was mucin-producing carcinomas, along with thrombin and fibrin, which might trigger these thrombotic phenomena. Some studies mentioned that a high PF4 level was associated with high thromboembolism risk in patients with pancreatic cancer (20, 21). For patients with pancreatic cancer and a high level of PF4, COVID-19 vaccination could be a risk of thromboembolism events. When an anti-PF4 antibody wad triggered by the COVID vaccine, the previous existed PF4 level in pancreatic cancer patients may result in more platelet activation and aggregation.

To our knowledge, this case study was the first to report a cancer patient who acquired VITT after mRNA-1273 vaccination. The information about the actual cancer-associated PF 4 and VITT was limited. Therefore, further studies regarding the VITT and cancer patients are necessary. The potential thromboembolism risk after COVID-19 vaccine administration should be considered for patients with cancer and a hypercoagulable state.

## Limitations

This is only the first case presented about a cancer patient and mRNA-1273 related VITT. The confounding factors including underlying disease were not well-controlled. We intend to do more analysis when multisource COVID-19 vaccination datasets were available.

## Data Availability Statement

The original contributions presented in the study are included in the article/supplementary material, further inquiries can be directed to the corresponding author.

## Ethics Statement

Ethical review and approval was not required for the study on human participants in accordance with the local legislation and institutional requirements. Written informed consent for participation was not required for this study in accordance with the national legislation and the institutional requirements. Written informed consent was obtained from the individual(s) for the publication of any potentially identifiable images or data included in this article.

## Author Contributions

Y-CY, W-HC, H-CL, and Y-TC performed the patient care and diagnosis. M-HC analyzed the laboratory diagnosis. P-HS and Y-MH wrote the first draft. All authors contributed to the article and approved the submitted version.

## Conflict of Interest

The authors declare that the research was conducted in the absence of any commercial or financial relationships that could be construed as a potential conflict of interest.

## Publisher's Note

All claims expressed in this article are solely those of the authors and do not necessarily represent those of their affiliated organizations, or those of the publisher, the editors and the reviewers. Any product that may be evaluated in this article, or claim that may be made by its manufacturer, is not guaranteed or endorsed by the publisher.

## Abbreviations

VITT: vaccine-induced thrombotic thrombocytopenia
PF4: platelet factor 4
CVST: cerebral venous sinus thrombosis.

## References

[1] Kuderer NM Choueiri TK Shah DP Shyr Y Rubinstein SM Rivera DR. Clinical impact of COVID-19 on patients with cancer (CCC19): a cohort study. Lancet. (2020) 395:1907–18. 10.1016/S0140-6736(20)31187-9 32473681 PMC7255743

[2] Corti C Crimini E Tarantino P Pravettoni G Eggermont AMM Delaloge S e al. SARS-CoV-2 vaccines for cancer patients: a call to action. Eur J Cancer. (2021) 148:316–27. 10.1016/j.ejca.2021.01.046 33770576 PMC7904467

[3] Schultz NH Sorvoll IH Michelsen AE Munthe LA Lund-Johansen F Ahlen MT. Thrombosis and thrombocytopenia after ChAdOx1 nCoV-19 vaccination. N Engl J Med. (2021) 384:2124–30. 10.1056/NEJMoa2104882 33835768 PMC8112568

[4] SARS-CoV-2 Vaccine-Induced Immune Thrombotic Thrombocytopenia. N Engl J Med. (2021) 384:e92. 10.1056/NEJMx210006 34110115

[5] Welsh KJ Baumblatt J Chege W Goud R Nair N. Thrombocytopenia including immune thrombocytopenia after receipt of mRNA COVID-19 vaccines reported to the vaccine adverse event reporting system (VAERS). Vaccine. (2021) 39:3329–32 10.1016/j.vaccine.2021.04.054 34006408 PMC8086806

[6] McMurry R Lenehan P Awasthi S Silvert E Puranik A Pawlowski C. Real-time analysis of a mass vaccination effort confirms the safety of FDA-authorized mRNA COVID-19 vaccines. Med (N Y). (2021) 2:965–78.e5. 10.1016/j.medj.2021.06.006 34230920 PMC8248717

[7] Schulz JB Berlit P Diener HC Gerloff C Greinacher A Klein C. COVID-19 vaccine-associated cerebral venous thrombosis in Germany. Ann Neurol. (2021) 90:627–39. 10.1101/2021.04.30.21256383 34288044 PMC8427115

[8] Krzywicka K Heldner MR Sánchez van Kammen M van Haaps T Hiltunen S Silvis SM. Post-SARS-CoV-2-vaccination cerebral venous sinus thrombosis: an analysis of cases notified to the European Medicines Agency. Eur J Neurol. (2021) 28:3656–62. 10.1111/ene.15029 34293217 PMC8444640

[9] Sangli S Virani A Cheronis N Vannatter B Minich C Noronha S. Thrombosis with thrombocytopenia after the messenger RNA-1273 vaccine. Ann Intern Med. (2021). 10.7326/L21-0244. [Epub ahead of print]. 34181446 PMC8251935

[10] Waqar SHB Khan AA Memon S. Thrombotic thrombocytopenic purpura: a new menace after COVID bnt162b2 vaccine. Int J Hematol. (2021) 114:626–9. 10.1007/s12185-021-03190-y 34264514 PMC8280631

[11] Ruhe J Schnetzke U Kentouche K Prims F Baier M Herfurth K. Acquired thrombotic thrombocytopenic purpura after first vaccination dose of BNT162b2 mRNA COVID-19 vaccine. Ann Hematol. (2021). 10.1007/s00277-021-04584-y. [Epub ahead of print]. 34309715 PMC8311064

[12] Al-Ahmad M Al-Rasheed M Shalaby NAB. Acquired thrombotic thrombocytopenic purpura with possible association with AstraZeneca-Oxford COVID-19 vaccine. EJHaem. (2021). 10.1002/jha2.219. [Epub ahead of print]. 34226899 PMC8242544

[13] Yocum A Simon EL. Thrombotic thrombocytopenic purpura after Ad26.COV2-S vaccination. Am J Emerg Med. (2021). 10.1016/j.ajem.2021.05.001. [Epub ahead of print]. 33980419 PMC8095021

[14] American Society of Hematology. Thrombosis with Thrombocytopenia Syndrome (also termed Vaccine-induced Thrombotic Thrombocytopenia). Available online at: https://www.hematology.org/covid-19/vaccine-induced-immune-thrombotic-thrombocytopenia?fbclid=IwAR28lsq1WJRrQP_lRuI6upWah9FO3_EQ54p352k0Qbl_Nw4qzLaD5d7QILk, (accessed August 12, 2021).

[15] Scully M Singh D Lown R Poles A Solomon T Levi M. Pathologic antibodies to platelet factor 4 after ChAdOx1 nCoV-19 vaccination. N Engl J Med. (2021) 384:2202–11. 10.1056/NEJMoa2105385 33861525 PMC8112532

[16] Novak N Tordesillas L Cabanillas B. Adverse rare events to vaccines for COVID-19: From hypersensitivity reactions to thrombosis and thrombocytopenia. Int Rev Immunol. (2021). 10.1080/08830185.2021.1939696. [Epub ahead of print]. 34251972 PMC8290371

[17] Rizk JG Gupta A Sardar P Henry BM Lewin JC Lippi G. Clinical characteristics and pharmacological management of COVID-19 vaccine-induced immune thrombotic thrombocytopenia with cerebral venous sinus thrombosis: a review. JAMA Cardiol. (2021). 10.1001/jamacardio.2021.3444 34374713

[18] Rock G Weber V Stegmayr B. Therapeutic plasma exchange (TPE) as a plausible rescue therapy in severe vaccine-induced immune thrombotic thrombocytopenia. Transfus Apher Sci. (2021) 60:103174. 10.1016/j.transci.2021.103174 34088601

[19] Varki A. Trousseau's syndrome: multiple definitions and multiple mechanisms. Blood. (2007) 110:1723–9. 10.1182/blood-2006-10-053736 17496204 PMC1976377

[20] Poruk KE Firpo MA Huerter LM Scaife CL Emerson LL Boucher KM. Serum platelet factor 4 is an independent predictor of survival and venous thromboembolism in patients with pancreatic adenocarcinoma. Cancer Epidemiol Biomarkers Prev. (2010) 19:2605–10. 10.1158/1055-9965.EPI-10-0178 20729288 PMC2952057

[21] Fiedler GM Leichtle AB Kase J Baumann S Ceglarek U Felix K. Serum peptidome profiling revealed platelet factor 4 as a potential discriminating Peptide associated with pancreatic cancer. Clin Cancer Res. (2009)15:3812–9. 10.1158/1078-0432.CCR-08-2701 19470732

